# How to interpret core concepts in POPIA? Recommendations on the draft Code of Conduct for Research

**DOI:** 10.17159/sajs.2023/15062

**Published:** 2023-08-08

**Authors:** Donrich Thaldar, Aliki Edgcumbe, Dusty-Lee Donnelly

**Affiliations:** 1 School of Law, University of KwaZulu-Natal, Durban, South Africa; 2 Petrie-Flom Center for Health Policy, Biotechnology, and Bioethics, Harvard Law School, Cambridge, Massachusetts, USA

**Keywords:** research code of conduct, consent, special personal information, responsible party, de-identification

## Introduction

The publication of the draft Code of Conduct for Research^1^ (draft CCR) by the Academy of Science of South Africa (ASSAf) in September 2022 is a welcome development. Overall, the draft CCR promises to be a useful document for the South African research community. It is written in plain language, contains useful diagrams and user-friendly hyperlinked cross-references, examples in the research context, and references to additional resources.

In terms of the draft CCR’s substance, the most striking positive element is how it deals with the concept of *public interest*. Public interest is a central concept in the *Protection of Personal Information Act 4 of 2013* (POPIA), and is important for research, as it is relevant when considering a research exception for allowing the processing of special personal information (section 27(1)(*d*)(*i*)), and a research exemption from the conditions for processing personal information (section 37(1)(*a*)). The Information Regulator has proffered a ‘basic formulation’ of public interest in a *Guidance Note*^2^, but this ‘basic formulation’ has been critiqued in the literature as misaligned with South African case law on public interest^3^. To ASSAf’s credit, the draft CCR (Table 5, page 24) does not simply follow the *Guidance Note*’s embattled ‘basic formulation’, but adopts a more pragmatic – and legally justifiable – meaning of public interest.

However, there is still a need to improve the way that the draft CCR deals with four other core concepts in POPIA, namely (1) consent; (2) special personal information; (3) responsible party; and (4) de-identification. In this article, we explain why there is a need for improvement of the draft CCR in the case of each of these concepts, and we make recommendations on how to improve the draft CCR in this regard.

## Consent

The interpretation of the meaning of *consent* in POPIA has been the subject of academic debate; members of our research group contended that consent, for purposes of POPIA, must be *specific*, as clearly provided for in section 1 of POPIA.^4–6^ However, there have been other scholars who have argued for an interpretation of consent in POPIA as meaning *broad* consent.^7^ Also, we have contended that POPIA constitutes a new layer of legal rules, and that POPIA compliance should therefore be differentiated from ethics compliance, as these are two distinct sets of rules, and the one does not subsume or replace the other.^4–6^ In this light, we commend ASSAf on confirming this law/ethics distinction in the draft CCR, and on making it clear that consent in POPIA must be *specific* – at least in the context of an *initial* research project.

However, we must raise concern about the following statement in the draft CCR that relates to *future* research (Table 4, page 20)^1^:

POPIA Consent for future use is allowed as long as the future uses of the Personal Information are not speculative, are described as fully as possible, and further use of the Personal Information is restricted.

Our concern has two parts. First, this statement in the draft CCR departs from POPIA. There is a difference between having a specific purpose and having a purpose that is merely ‘not speculative’. Accordingly, this risks watering down the requirement of *specific* consent, and calls for serious reflection. Second, given POPIA’s research exceptions (in sections 15(3)(*e*) and 27(1)(*d*)), consent for *future* research projects is *not* necessarily a POPIA requirement. In other words, from the perspective of POPIA, researchers may rely on consent for further processing, but they do not have to. Accordingly, we recommend that the statement in the draft CCR pertaining to consent for *future* research should be removed. From the perspective of POPIA, it is both problematic and unnecessary.

However, depending on the circumstances, consent to a further research project may be required by the institutional research ethics committee. This raises the question: how should researchers integrate the POPIA and ethics requirements regarding consent at the stage of collecting information from research participants? In brief, for the *initial* research project, consent must be for a *specific* purpose, as required by section 13(1) of POPIA. Researchers may include additional provisions if required by institutional research ethics committees. As an example, consider the question: ‘May we contact you again for a future research project or follow up on your responses?’ Such a provision is self-evidently only an ethical consideration. From a legal perspective it is not consent to future research, much less *specific* consent. At the same time, it may also be wise to request research participants to provide consent now for *future* research. The mode of consent that is appropriate – e.g. specific, tiered, or broad consent – is determined by the relevant institutional research ethics committee, and is an ethics requirement, not a POPIA requirement. It is essential to approach this enquiry from a pragmatic perspective. One can readily imagine the futility of trying to predict all the uses of data that may become possible in the light of advancing data science techniques. It is precisely for these kinds of situations that we recommend researchers familiarise themselves with POPIA’s research exemptions. ASSAf may consider including this as a consolidated legal-ethical note for clarification.^8^

## Special personal information

POPIA broadly regulates two types of information: personal information and special personal information, which are each defined in POPIA. Importantly, special personal information is defined with reference to personal information (in section 1 of POPIA) as follows: ‘“special personal information’ means personal information as referred to in section 26’. Ergo, special personal information is a *subclass* of personal information. Logic dictates that provisions in POPIA that apply to personal information would *include* special personal information *qua* subclass of personal information, unless special personal information is specifically excluded.

Now consider POPIA’s processing requirements. In respect of *personal information*, section 11 provides that at least one of the legal grounds for processing personal information listed in that section must be present. In respect of *special personal information*, section 26 provides that if personal information qualifies as special personal information, it may not be processed – except if at least one legal ground for processing special personal information listed in section 27 is present. Importantly, given that special personal information is a subclass of personal information, special personal information is not exempted from compliance with section 11. Accordingly, sections 26 and 27 of POPIA apply as an *extra layer* of rules over the basic provisions of section 11.9 Stated differently, sections 26 and 27 are *not* an *alternative* compliance pathway for special personal information; they constitute an *additional* compliance pathway for special personal information.

However, the draft CCR (paragraph 4.3.3.3.5, page 19) states that ‘Any of the following legal justifications [referring to the grounds listed in section 11 of POPIA] must apply when the Research does NOT include Special Personal Information.’ This has the effect of exempting special personal information from compliance with section 11. We suggest that this is a mistake that should be corrected. Moreover, it would assist the research community if a *general* provision could be included in the draft CCR that clarifies that special personal information is a subclass of personal information that must comply with an *extra layer* of requirements. This is important, as the issue is not only relevant to the processing limitations in POPIA, but also to further processing and the research exceptions (section 15(3)(*e*) for personal information and section 27(1)(*d*)(*i*) for special personal information).

## Responsible party

Section 1 of POPIA defines a responsible party as ‘a public or private body or any other person which, alone or in conjunction with others, determines the purpose of and means for processing personal information’. Accordingly, who qualifies as a responsible party is a question of fact: who determines the purpose of, and means for, processing personal information? In the research context, a responsible party is likely to include both (1) individual researchers; and (2) research institutions – i.e. juristic persons that conduct research.^10^ Importantly, no juristic person can ever determine the purpose of, and means for, processing personal information on its own – there must always be at least one individual who makes this determination.^10^ We acknowledge that it may be possible, pragmatic even, to proceed from the standpoint that in a typical employment relationship the employee merely acts as an agent of their employer. But we warn that it is dangerous to assume that this will always be the case.

In research it is the principal investigator who will exert primary control over determining the purpose and means of processing. In our view, researchers and their employing research institutions would all be ‘responsible parties’ as defined in section 1 of POPIA. Importantly, this statutory definition cannot be narrowed by a code of conduct if that would amount to amending the provisions of the statute.^10^ However, that is exactly what the draft CCR purports to do. It proposes that a responsible party does *not include* an individual researcher if such a researcher is in the employ of a research institution. In other words, irrespective of the *facts* of who actually determines the purpose of, and means for, processing personal information, the draft CCR proposes that only the juristic person *qua* employer should be legally liable for POPIA compliance. This is a narrowing of the statutory definition provided for in POPIA and is clearly not permissible.

The draft CCR states (Table 11, page 61): ‘An entity acting through its employees is vicariously liable for the actions of those employees, provided that the employee is acting within the course and scope of their employment.’ This is true, but it does not mean that the employee is excluded from liability. Vicarious liability is a legal concept adopted in the branch of law that imposes liability for causing harm to another (known in South Africa as *delict*). Where the person who caused the harm was an employee acting within the course and scope of their employment, then the law holds *both* the employee and the employer jointly and severally liable. Importantly, vicarious liability thus does not exclude the employee from liability. The injured party is free to choose whether they will sue the employee, the employer or both. Vicarious liability is thus a tool in the hand of a *plaintiff* to choose who to sue; it is *not a defence* in the hand of an employee. In the context of POPIA and research, the plaintiff will be a *research participant* (or to use POPIA terminology, a *data subject*) who intends to sue for damages in terms of section 99. As a general rule in our law, a plaintiff has a procedural *right to choose who to sue* among potential defendants.^10,11^ The plaintiff therefore has the right to choose to sue: (1) the individual researcher who determined the purpose of, and means for, processing personal information; or (2) the juristic person that employed the individual researcher (based on vicarious liability); or both (1) and (2).

Accordingly, if vicarious liability is properly understood, it is clear that it cannot serve as a rationale for narrowing POPIA’s definition of responsible party. Quite the opposite – it highlights that the draft CCR’s proposed exclusion of individual researchers from qualifying as responsible parties, if such researchers are in the employ of research institutions, will infringe on the legal procedural rights of data subjects, and hence compromise their right to access the courts, protected by section 34 of the South African Constitution.^12^

Consider the following hypothetical facts: Professor X is the principal investigator of a research project on HIV. Professor X writes a research protocol that provides for the collection of biospecimens and health information from people living in a local community. The protocol sets out the purpose of, and means for, processing the data generated from studying the biospecimens and the health information collected directly from the research participants. Professor X is in the employ of University Y. The research protocol is approved by University Y’s health research ethics committee. However, after all the data have been generated and collected, Professor X materially fails to comply with POPIA. As a result, there is a data breach and the identities of the research participants, who are HIV positive, become public knowledge. Z, one of the research participants who has been identified as being HIV positive, is ostracised by his community and loses his work. Z’s legal aid attorney writes a letter to both Professor X and University Y, requesting a meeting in an attempt to settle the matter in a non-litigious fashion. The meeting takes place and University Y’s representatives offer a sincere apology to Z. They also agree to issue a public apology. Z accepts this offer from University Y. However, Professor X refuses. She states that people must learn to be open about their HIV status, and leaves the meeting. Z has the right to choose to sue Professor X alone for damages. Any attempt to deny Z this right would infringe on Z’s constitutional right of access to justice.

Clearly, the draft CCR’s proposed exclusion of individual researchers from qualifying as responsible parties, if such researchers are in the employ of research institutions, is not only misaligned with POPIA, but is also on shaky constitutional ground. We suggest that ASSAf rethink this issue. Instead of attempting to extract individual researchers from litigation, which is not legally possible, a better way to ameliorate the position of individual researchers in the face of strict liability litigation is for research institutions to indemnify their own employees against such litigation.^10^ This approach can be *recommended* in the draft CCR, and each research institution can decide whether, and how, to implement it. While indemnification will not extract individual researchers from the litigation process, it will ensure that they are not personally bankrupted by their legal costs and a potential damages award.

## De-identification

The use of foreign terminology in the draft CCR, especially the use of *anonymisation* as a synonym for POPIA’s reference to *de-identification*, is problematic. While *anonymisation* and *de-identification* are used in other jurisdictions and frequently appear in biomedical literature, the concepts are distinct and often defined in conflicting ways, both within statues and the literature.^13^ Because there is no global consensus on the meaning and use of the terms *de-identification* and *anonymisation*, particular care is needed when employing them. Therefore, we recommend that these terms should not be used synonymously in the draft CCR; instead, they should be explained to clarify how POPIA’s *de-identification* differs from corresponding terms found in other jurisdictions. The correct term in South Africa is *de-identification* – and it has been given a distinct definition in section 1 of POPIA, where it means

to delete any information that— (a) identifies the data subject; (b) can be used or manipulated by a reasonably foreseeable method to identify the data subject; or (c) can be linked by a reasonably foreseeable method to other information that identifies the data subject.

For information to be de-identified, and excluded from the scope of POPIA, section 6(1)(*b*) provides that the information must be de-identified ‘to the extent that it cannot be re-identified’. The test employed is unique to South Africa – and quite stringent: if any information can be ‘used’, ‘manipulated’ or ‘linked’ by ‘a reasonably foreseeable method’ to re-identify the data subject, it is not de-identified information in terms of POPIA (section 1 of POPIA).

Although the term *de-identification* is also used in the USA, the concept (and its meaning) differs from that in POPIA. Within the *Health Insurance Portability and Accountability Act of 1996* (HIPAA), de-identification relies on applying one of two methods: (1) the removal of 18 personal identifiers from a data set, with the important proviso that the researcher has no ‘actual knowledge’ that the residual information may identify an individual (the *Safe Harbor* method, §164.514(b)(2)); or (2) a determination made by a suitably qualified expert who must establish that the risk of re-identification ‘is very small’ (the *Expert Determination* method, §164.514(b)(1)).^14^ Both methods speak to the overarching principle that, to be de-identified, the information ‘does not identify an individual and… there is no reasonable basis to believe that the information can be used to identify an individual’ (§164.514(a)). Notably, a covered entity may still assign a code or other means of record identification to allow de-identified information to be re-identified at a later stage (§164.514(a)), whereas this would not be permitted under POPIA’s definition of de-identification.

The term *de-identification* is also used in the United Kingdom’s *Data Protection Act 2018* (DPA). Section 171(1) of the DPA provides that it is an offence to ‘knowingly and recklessly re-identify information that is de-identified personal data’. It is in this context that section 171(2)(a) of the DPA provides that ‘personal data is “de-identified” if it has been processed in such a manner that it can no longer be attributed, without more, to a specific data subject’. Therefore, de-identification in the context of HIPAA and the DPA is clearly not equivalent to de-identification in POPIA.

Furthermore, although *pseudonymisation* is used within the draft CCR, it is not a concept found in POPIA. Rather, it appears in other international instruments such as the European Union’s General Data Protection Regulation 2016/679 (GDPR) where, in Article 4(5), pseudonymisation is described as the process of storing additional information separately that can be used to re-identify the data subject.^15^ As re-identification is possible in the case of pseudonymised data, the data continue to be considered personal data and must be treated as such. Thus, data that can be re-linked using a code, algorithm or pseudonym remain personal data under the GDPR, and the position is the same under POPIA. Although POPIA does not expressly refer to the term, it remains an important data privacy safeguard, and section 19 is broad enough to encompass pseudonymisation of data in its requirement that ‘appropriate, reasonable technical and organisational measures’ are used to secure the privacy of data subjects.

For this reason, the draft CCR should avoid the use of terms that clearly have different standards and definitions to what is required by POPIA. Instead, it should be made clear that *de-identification* in South Africa is not equivalent to corresponding terms employed in other jurisdictions and that the test for de-identification is unique to South Africa. We recommend that ASSAf clarifies the South African position to resolve any confusion around the use of these terms.

## Conclusion

The draft CCR is breaking new ground in a relatively new field of the law. If approved by the Information Regulator, the draft CCR will – for the duration of its 5-year lifecycle – become an important document for the South African research community. Accordingly, before submitting the draft CCR to the Information Regulator, it is essential that ASSAf irons out the issues that we have identified in this article – especially because these issues relate to four core concepts in POPIA. Our recommendations are summarised in Table A.

## Figures and Tables

**Table A:** Recommendations for deletions, alterations, and inclusions in the draft CCR

Core concept	Recommendations
Consent	*Key recommendation:* Remove the explanation relating to *consent* for future research. Table 4, page 20, delete: *Further recommendation:* For clarity, ASSAf may consider providing a consolidated legal-ethical note, which explains that the mode of consent for *future* research is determined by the relevant institutional research ethics committee.
Special personal information	*Key recommendation:* Clarify that *special personal information* is a subclass of *personal information* that must comply with an *extra layer* of requirements. We recommend that ASSAf include a *general* provision in the draft CCR that clarifies this. Paragraph 4.3.3.3.5 and Table 4, page 19, amend: ‘Any of the following legal justifications [referring to the grounds listed in section 11] must apply to the processing of Personal Information’.Paragraph 4.3.3.3.5, page 19, recommended wording of the suggested inclusion: Note: Special Personal Information is a subclass of Personal Information. When Research includes Special Personal Information, it must comply with an extra layer of requirements. [See Table 5 ].’
Responsible party	*Key recommendation:* Do not exclude individual researchers employed by research institutions and public bodies from qualifying as *responsible parties*. Page 7, paragraph 2.2.2, amend: ‘The private or Public Body that employs, or directly controls the researchers will be the Responsible Party , along with the researcher(s) who determine(s) how and why personal information is processed.Page 7, paragraph 2.2.2, delete: Table 11, page 61, delete: *Further recommendation:* The draft CCR may include a recommendation that public bodies and research institutions indemnify their own employees against litigation.
De-identification	*Key recommendations:* • Throughout the draft CCR, clearly differentiate *de-identification* in the South African context from corresponding terms used in other jurisdictions. • Delete the terms *anonymise/anonymised/anonymisation* when used as a synonym for de-identification. (For example, see paragraph 1.1.2.2.1, page 5, and paragraph 1.3, page 64.) • Explain references to foreign terms and documents. (For example, see Table 9, page 40.) • Remove references to foreign tests (used in the draft CCR to determine whether data is de-identified). The test within POPIA is unique. (For example, see paragraph 1.7, page 65, and Table 1, page 72.) *Further recommendations:* We recommend that the use of foreign terms should be avoided, as these clearly have different definitions, standards, and tests to what is required by POPIA. Where they are employed, it should be made clear that *de-identification* in POPIA is not equivalent to corresponding terms used in other jurisdictions and that the test for de-identification is unique to South Africa.

## Significance:

The draft Code of Conduct for Research is a welcome development, but there is room for improvement in the way that it interprets core concepts in POPIA. In particular, it should: remove the provision regarding *consent* to future research; clarify that *special personal information* is a subclass of personal information that must comply with an extra layer of requirements; not exclude an individual researcher employed by a research institution from qualifying as a *responsible party*; and clearly differentiate *de-identification* in POPIA from corresponding terms used in other jurisdictions.
